# p62-DNA-encoding plasmid reverts tumor grade, changes tumor stroma, and enhances anticancer immunity

**DOI:** 10.18632/aging.102486

**Published:** 2019-11-21

**Authors:** Franco M. Venanzi, Vladimir Gabai, Francesca Mariotti, Gian Enrico Magi, Cecilia Vullo, Albert A. Sufianov, Sergey I. Kolesnikov, Alexander Shneider

**Affiliations:** 1 Sechenov First Moscow State Medical University, Moscow, Russia; 2 CureLab Oncology, Inc, Deadham, MA 02026, USA; 3 Department of Biochemistry, Boston University School of Medicine, Boston, MA 02118, USA; 4 School of Biosciences and Veterinary Medicine, University of Camerino, Camerino, Italy; 5 Russian Academy of Sciences, Moscow, Russia; 6 Lomonosov Moscow State University, Moscow, Russia; 7 Research Center of Family Health and Reproduction Problems, Irkutsk, Russia; 8 Department of Molecular Biology, Ariel University, Ariel, Israel; 9 Federal Center of Neurosurgery, Tyumen, Russia

**Keywords:** canine tumors, p62 /SQSTM1, DNA plasmid, tumor microenvironment, anti-cancer immunity

## Abstract

Previously, we reported that the administration of a p62/SQSTM1-encoding plasmid demonstrates high safety and signs of clinical benefits for human cancer patients. The treatment also suppressed tumor growth and metastasis in dogs and mouse models. Here we investigated some mechanistic aspects of these effects. In mammary tumors bearing-dogs, i.m. injections of p62 plasmid reduced tumor sizes and their aggressive potential in 5 out of 6 animals, with one carcinoma switching to adenoma. The treatment increased levels of smooth muscle actin in stroma cells and type III collagen in the extracellular matrix, which correlate with a good clinical prognosis. The p62 treatment also increased the abundance of intratumoral T-cells. Because of the role of adaptive immunity cannot be tested in dogs, we compared the protective effects of the p62 plasmid against B16 melanoma in wild type C57BL/6J mice versus their SCID counterpart lacking lymphocytes. The plasmid was only protective in the wild type strain. Also, p62 plasmid amplified the anti-tumor effect of T-cell transfer from tumor-bearing animals to animals challenged with the same tumors. We conclude that the plasmid acts via re-modeling of the tumor microenvironment, making it more favorable for increased anti-cancer immunity. Thus, the p62-encoding plasmid might be a new adjuvant for cancer treatments.

## INTRODUCTION

Recent advances in cancer immunotherapy, particularly immune checkpoint blockade therapy, have dramatically changed the therapeutic strategy against advanced malignancies [1]. Yet, only a subset of patients demonstrates a positive response to such therapy. Moreover, questions relating to how we can maintain durable clinical responses or how we can successfully treat a broader range of cancers by immunotherapy remain largely unsolved.

Growing evidences suggest that the major barrier to more successful cancer immuno /chemotherapy is the tumor microenvironment (TME), where chronic inflammation has a predominant role in tumor survival and proliferation, angiogenesis and immunosuppression [2–5]. Since our understanding of cancer-related inflammation has significantly evolved, we have now various therapeutic options tailored to the TME [6]. These therapeutic strategies include inhibiting inflammatory mediators or their downstream signaling molecules, blocking the recruitment of myeloid cells, modulating immunosuppressive functions in myeloid cells and reeducating the TME [7].

So far, no conclusive studies have been published on stromal content and prognosis in human breast cancer [8]. In an effort to integrate the effects of the TME and patient outcome into pathological criteria, it has been reported that a “reactive” stromal phenotype may predict breast cancer subtypes with an excellent prognosis [9].

Accordingly, the lowest risk tumors were more likely to have high intra-tumoral stromal volume-density and high expression of stromal proteins, including alpha-smooth muscle actin (*alpha*-SMA), an actin isoform that marks myofibroblasts and cancer associated fibroblasts. Collagens are also critical components of the extracellular matrix (ECM) that regulates tumor progression. Although most research on collagen in breast cancer was focused on type I collagen (Col 1) and its pro-carcinogenic effects [10–12], new evidence suggest that a related fibrillar type III collagen (Col 3) plays an important role in suppressing primary tumor growth and metastasis in a murine model of triple - negative breast cancer [13, 14]. However, the role of Col 3 as a co-stimulatory molecules for lymphocytes has not been investigated. While ECM represents a physical barrier to immune cell infiltration, it also provides the substratum essential to the interstitial migration of immune cells [15, 16].

The role which p62/SQSTM1 plays in cancer and tumor stroma cells is a subject of active research [17–20]. A human p62-encoding plasmid was originally proposed as a classic DNA vaccine eliciting adaptive immune response against the p62/SQSTM1 protein over-expressed in cancer cells [21, 22]. However, the history of science and medicine bears multiple examples when a new phenomenon was explained based on the mechanisms which were most popular at the time they were observed, later turning out to be secondary or insignificant [23]. Thus, the mechanism of action of the p62 plasmid needs to be reassessed based on the latest observations. Indeed, although treatment with the p62 - encoding plasmid was reported as therapeutically beneficial in dogs with spontaneous mammary tumors [24], it turned out later that unlike in humans, most aggressive canine breast tumors show very low or nil p62 expression [25]. In other word, eliciting an anti-p62 adaptive immunity cannot be the only effect of p62-encoding plasmid.

We have already shown that the p62 DNA treatment dramatically impacted the histopathological characteristics of the malignant lesions. Indeed, following p62 DNA injections, the original solid tumors appear now as multi-lobate neoplasms, separated and surrounded by thick bands of inflamed fibrous connective, containing scattered aggregates of macrophages, increased number of CD3+ intratumoral T-lymphocytes (TIL), and plasma cells. [24]. Taken together, this data raises the question of whether p62 plasmid can alter the TME in a way favorable for anti-cancer immune response.

Another line of research revealed that administering the p62 plasmid reduces systemic chronic inflammation in rodent models resulting in the prevention and / or alleviation of a number of diseases. For example, the plasmid reduced metabolic syndrome induced by a high calorie high fat Western diet [26] an effect on this disease that may be linked to anticancer effects [27]. Osteoporosis is also a disorder pertaining to chronic inflammation which shares common signaling pathways with cancer (e.g., RANK/RANKL signaling) [28]. The p62 plasmid demonstrated both preventive and therapeutic effects in a mouse model of ovariectomy-induced osteoporosis with an effect on pro-inflammatory cytokines and RANK/RANKL signaling [29]. Also, p62 DNA has been reported to delay the development of age-related macular degeneration (AMD) in rapidly aging OXYS rats [30], which may constitute another example of the an anti - inflammatory effects.

Like in humans, canine spontaneous mammary carcinomas are very heterogeneous in terms of morphology and biological behavior [31]. Simple and complex carcinomas are recorded as the most common types of mammary malignances [32, 33]. Solid and tubulopapillary mammary carcinomas reveal both histological and molecular homology to human breast carcinomas [34]. Of note, in both canine mammary tumors and in human prostate cancer, the number of intratumoral T-lymphocytes is reported to be higher in benign lesions than in their malignant counterparts [35, 36]. Thus, testing the effect(s) of a cancer treatment on canine model may provide valuable comparative oncology clues.

## RESULTS

### Anti-tumor activity of p62 DNA & histopathological features

The anti-tumor effects of p62 DNA has been evaluated in a cohort of six (6) dogs bearing *simple* mammary tumors (Table 1). As shown in Table 2, the new trial confirms previous results [24] with p62 DNA-treated patients showing a marked reduction of the sizes of their neoplastic masses without complete tumor eradication. Next, microscopic examinations revealed that p62 DNA induced a remarkable infiltration of inflammatory / immunocells in the original tumors, never observed in untreated dogs, nor in patients injected with the irrelevant (sham) pcDNA3.1 plasmid (See Supplementary Figure 1A, 1B). Histopathological changes observed after p62DNA injections, are summarizes in Table 3. Two patients (# 4 and #6) demonstrated the switching of their tumor histotypes from simple high malignant lesions to less aggressive complex carcinoma. In one patient (#5), a low malignant tubulo-papillary carcinoma reverted to adenoma. Patient #1 showed a transition from solid to tubulo-papillary histotype, while in patient (# 3) the residual tumor maintained the same histotype and grade as that of the original lesion. Importantly, all p62-treated patients, are still tumor and metastasis-free and maintain good quality of life 4 years after surgery (mastectomy).

**Table 1:** Patients characterization.

**Pt#**	**Breed**	**Age (yrs)**	**Type**	**WHO Stage**
1	German Seph.	11	SCa	T3-N0-M0
2	Mongrel	10	TP Ca	T1-NO-MO
3	German Seph.	8	SCa	T3-N2-M1
4	Poodle	14	SCa	T2-N1-M0
5	Breton	10	TP Ca	T1-N0-M0
6	Boxer	10	TP Ca	T2-N0- MO

Lesion types: SCa, solid carcinoma; TPCa, Tubo-papillary carcinoma.

**Table 2:** p62 DNA antitumor activity.

**Tumor size (cm)**						
**Pts #**	**Dose × injection (mg)**	**# injections**	**Initial**	**Final**	**Change Tumor volum. (%)**	**Mastectomy**
1	0.75	9	8,6 × 6.2	6,4 × 3,4	−78	YES
2	0.75	3	1,1 × 0,7	0,5 × 0,6	−66	YES
3	1.5	9	20 × 16	19× 12	−75	YES
4	1.5	3	2,7 × 3,5	2,7 × 2,7	−40	YES
5	0.75	9	4,9 × 3,5	3×3	−55	NO
6	0.75	9	3,2 × 1,8	2×2	−23	YES

**Table 3:** Tumor histotypes / grades before and after p62 DNA treatment.

**Pts#**	**Before**	**MS**	**After**	MS*
1	SC	+++	TP	++
2	TP	++	TP	+
3	SC	+++	SC	+++
4	SC	+++	CC	++
5	TP	+	TPA	
6	TP	+++	CC	+

TP: tubulo-papillary carcinoma; CC: *complex* carcinoma; TPA: tubulo - papillary adenoma.

*MS (Malignancy Score) as obtained by combining WHO Stage and grading 32: (+++), high; (++), middle; (+), low.

### p62 DNA up-regulates *alpha*-SMA and type III collagen expression

Upregulated expression of *alpha*- SMA and / or Col 3 is associated with a good prognosis in breast cancer [9, 13, 14].

**Figure 1:**
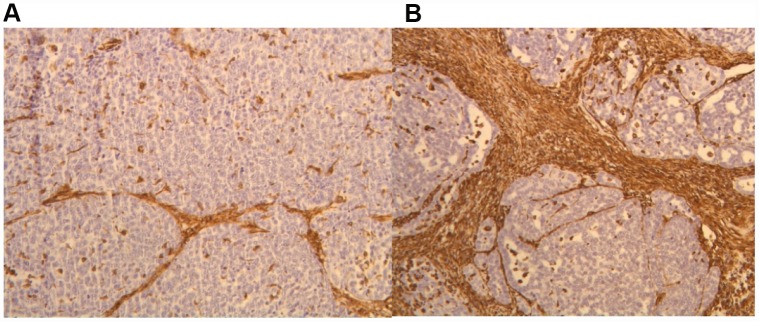
IHC staining of *alpha*- SMA in the tumor stroma before (**A**) and after (**B**) p62 DNA treatment. (20 ×).

**Figure 2:**
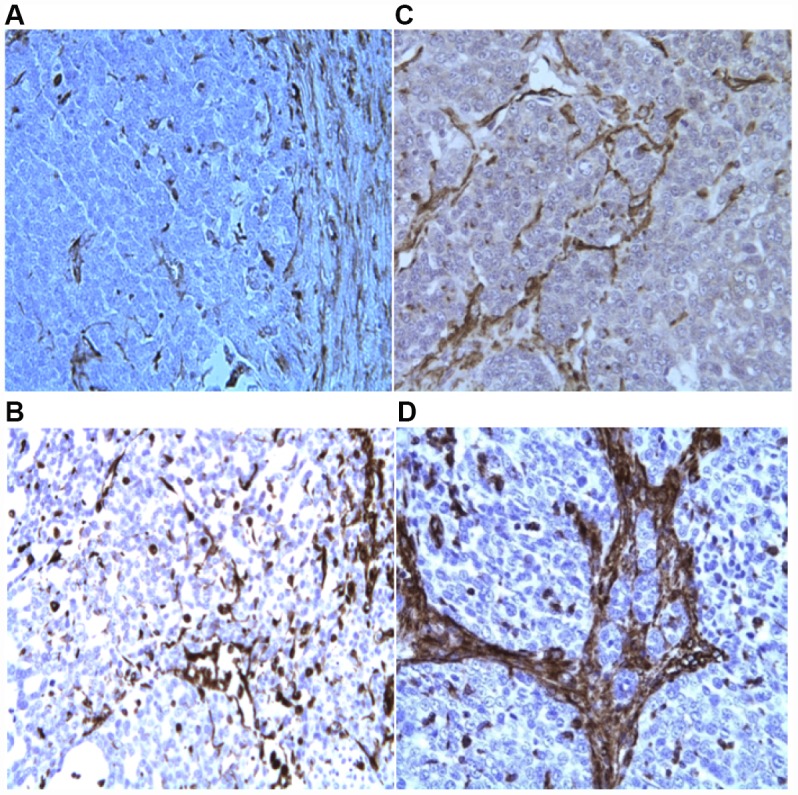
IHC evaluation of Col 1 (**A**, **C**) and Col 3 expression (**B**, **D**) in tumor biopsies, before (**A**, **B**) and after (**C**, **D**) p62 DNA injections (20×).

**Figure 3:**
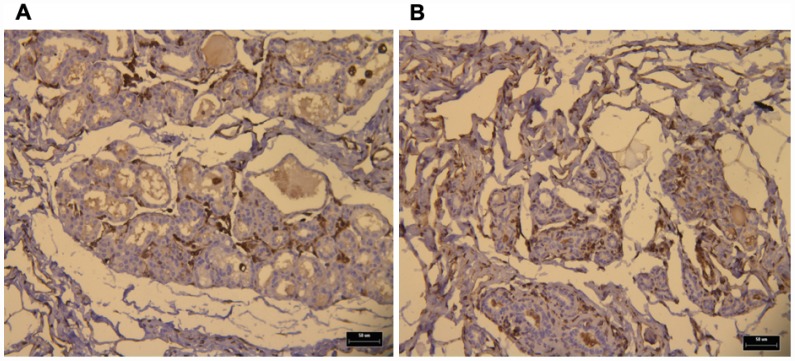
IHC of normal a mammary gland (NMG) showing both Col1 (**A**) and Col 3 (**B**) basal expression levels (brown dots). Bars, 50 μm.

We utilized immunohistochemistry to test if their levels change in response to p62 DNA treatment re-organizing the tumor microenviornement and making it more favorable to anti-cancer immunoresponse. Indeed we observed that p62 DNA administration induced strong increase in the expression of stromal *alpha*- SMA (Figure 1) coupled with a robust synthesis and deposition of Col 3 in the ECM, as opposed to a next to basal expression of Col 1 (Figure 2). On the other hand, both Col 1 and Col 3 levels are minimal in a normal mammary gland (Figure 3).

### The adaptive immune system is essential for the anti-cancer effect of p62 plasmid

We reported that in dogs p62 treatment increases the number of TILs [24]. However, in canine spontaneous tumors we cannot establish if an adaptive immune system is essential for the antitumor effect of p62 plasmid. Moreover, we know already that eliciting a specific anti-p62 immunity cannot be the only mechanism for the plasmid anti-cancer activity, since canine cancer cells (unlike human ones) omit p62 during tumor progression [25]. To bring these two facts together, we hypothesized that p62 potentiates the effects of T-cells targeting cancer antigens other than p62 (e.g., acting via immunomodulatory mechanisms). If that is correct, p62 plasmid would increase the efficiency of any other therapy generating T-cells targeting tumor associated antigens (e.g., immunotherapies).

**Figure 4:**
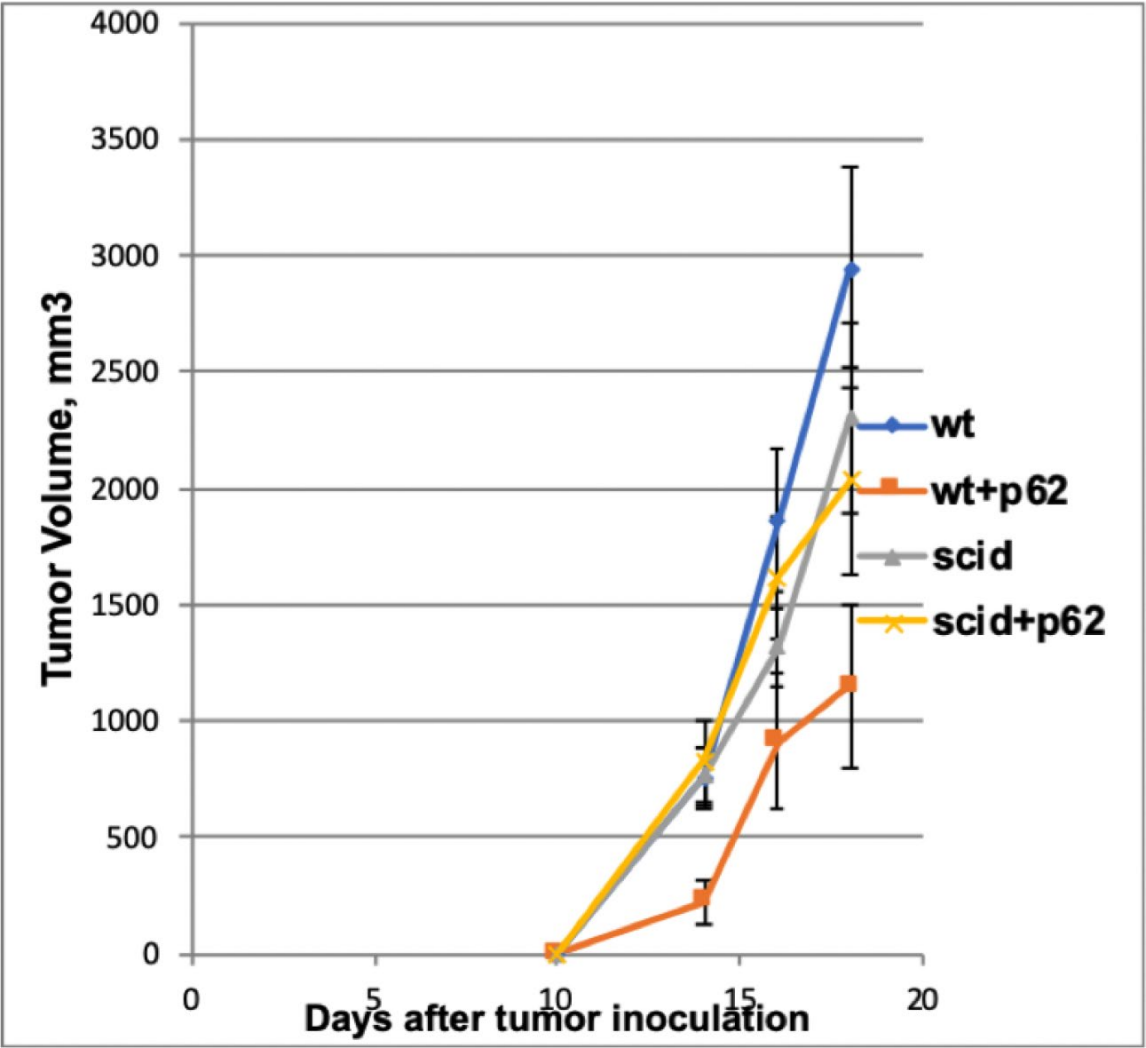
**Effect of p62 plasmid on the growth of B16 melanoma in Wt and immunodeficient (SCID) mice. While the p62 plasmid inhibited tumor growth in wt mice, no effect was found in immunodeficient mice.** Wt + p62 vs Wt: day14, p = 0.008; day16, p=0.02; day18, p =0.01 SCID +p62 vs SCID: day 14, p=0.40; day 16, p=0.31; day18, p=0.37.

**Figure 5:**
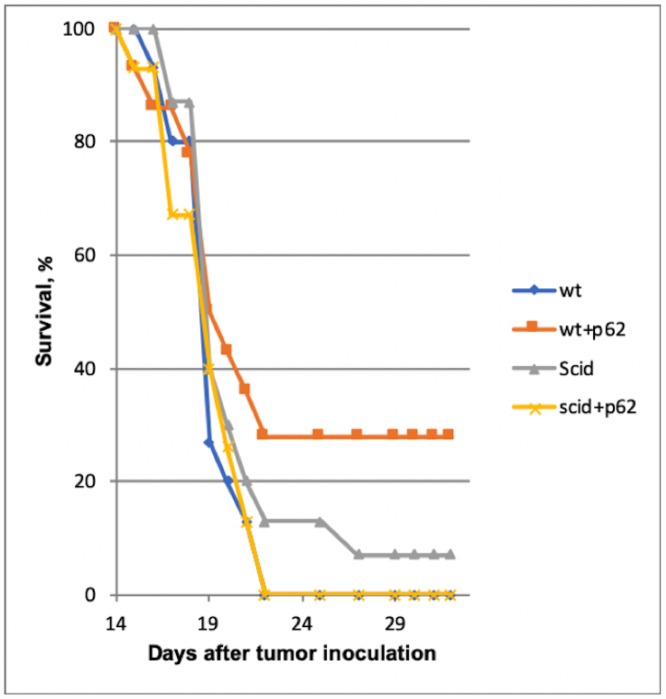
**Effect of p62 on the survival of Wt and immunodeficient (SCID) mice. Wt+p62 vs Wt p=0.026 at day 25.**

**Figure 6:**
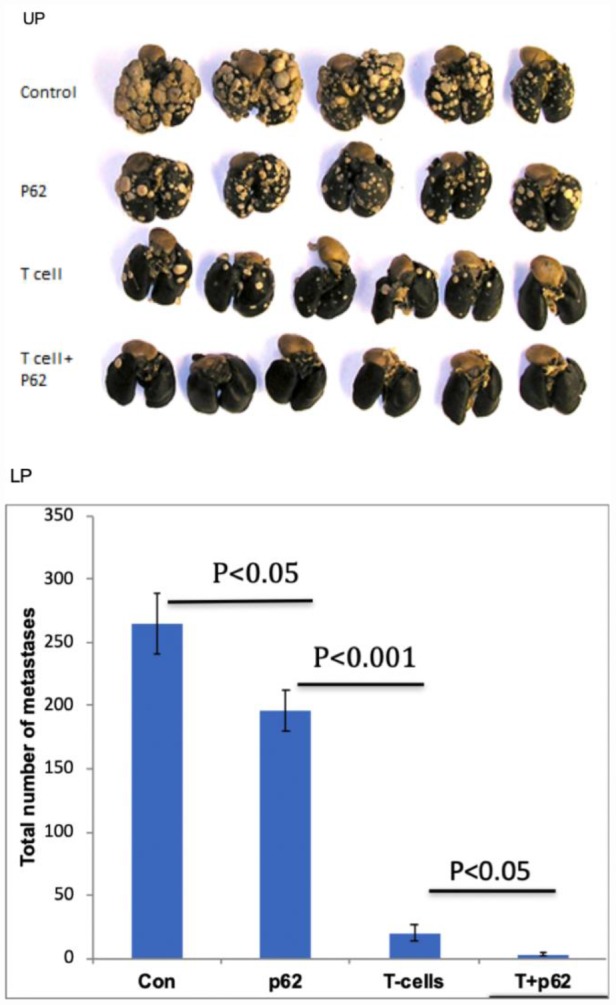
**Effect of p62 plasmid on adoptive T-cell transfer.** Upper panel (UP): lung metastases formed 26 days after i.v. injection of MCA205 fibrosarcoma cells; Lower panel (LP): results quantification.

The above presented data made two option equally plausible. The first hypothesis would state that p62 plasmid induce the re-organization of the tumor stroma, and this change(s) is sufficient for the antitumor effect of the plasmid. An alternative hypothesis would state that p62 plasmid acts primarly via adaptive immunity. If the first hypothesis is correct, the antitumor effects would be the same both in wild-type mice or in syngeneic mice with severe combined immunodeficiency (SCID) SCID mice have a genetically inactivated adaptive immune system (i.e. lacking T-and B-cells), but maintain an intact innate immune system (i.e., macrophages, NK cells etc). As depicted in Figure 4, when SCID mice were challenged with B16 melanoma cells, they developed tumors similar to control (wt) mice, indicating that the lack of an adaptive immune system does not promote tumor development in this model. However, in contrast to wt mice, the p62 plasmid lost its ability to inhibit tumor growth in SCID animals (Figure 4). Furthermore, whereas p62 DNA increased survival in wt mice, no such effect was seen in SCID mice (Figure 5). Thus, we conclude that an adaptive immune system is required for the anti-cancer of the p62 plasmid. Finally to test if the p62DNA can enhance the effects of other immunotherapies, we employed a model of adaptive cell transfer, where T-cells from tumor-bearing mice are transferred to animals with established tumor (or metastasis). In this model, the p62 plasmid was administered on days 9 and 14 after the animals received the transplantable tumor, one week before the mice were sacrificed. The days of plasmid administration left us time period to short to develop a protective anti-p62 immunity which could block / reduce the formation of lung metastasis. As shown in Figure 6, while p62DNA alone shows only a minor effect on lung metastasis, it amplified the anti-metastatic effects of transferred T-cells.

## DISCUSSION

The present translational oncological study was stimulated by the observations made during phase I / IIa clinical trials of patients with advanced ovarian and breast cancer. We reported transient increase in progression-free survival in a majority of patients, and a partially restored sensitivity to chemotherapy in all patients treated with the p62 plasmid [37]. Although the causes behind the development of drug resistance include different mechanisms [38], growing evidence indicates that changes in tumor microenvironment may contribute to resistance against chemo and radiotherapies [39]. It is a commonly accepted view that the lower the grade of a tumor is, the more sensitive the tumor would be to a therapy. The results of the present paper demonstrate that the treatment with the p62 DNA induces dramatic stromal alterations reverting a tumor grade towards less aggressive lesions. If the same phenomenon takes place in humans, it would explain why the p62-treated patients became responsive to chemotherapy.

As reported before [9], building up a “reactive” stromal phenotype characterized by alfa-SMA and Col 3 accumulation can constrain tumor growth. Our results (Figures 1–3) demonstrate that p62 DNA administration lead to increased intratumoral expression of both proteins.

Indeed, Col 3 expression levels were greatly increased in tumor ECM of treated dogs. At the same time, the fact that Col 1 was not upregulated to the same extent may not be too surprising. For example, it is known that matrix metalloproteinase cleaves Col I while Col 3 level increases [40]. Similarly, it was reported that TNF-alfa, a major pro-inflammatory cytokine, downregulates stromal alfa-SMA [40]. On the other hand, we previously demonstrated that p62 DNA quenches an ovariectomy-induced increase of TNF-alfa levels [29]. Thus, our observations support the hypothesis that p62 plasmid drives a stroma re-programming towards tumor suppression and increasing therapeutic vulnerability.

Remodeling of the ECM may partially explain the increased number of T- lymphocytes we observed in canine tumors following p62 plasmid treatment [24]. It was suggested that the trafficking and motility of T lymphocytes is guided by collagen fibers [41]. Thus, by creating a network of EMC fibers throughout the tumor, p62-DNA could promote axis of collagen alignment, which T cells can move along. According to a recently-stipulated hypothesis, ECM composition could define collective cooperated lymphocyte motility as opposed to an individual trafficking of the cells [42]. Although this hypothesis was suggested for B-lymphocytes only, it would be interesting to test if the same takes place for T-lymphocytes, and whether the p62 plasmid induces such collective lymphocyte tumor penetration.

Despite all the facts linking the extracellular matrix to TILs, it remained to be possible that treatment with the p62 plasmid acts on cancer cells through a mechanism not involving an adaptive immune response. For example, it was reported [13] that reduced Col 3 level in heterozygous mice led to increased tumor formation *in vivo* when the mice were challenged with transplantable breast cancer model. *In vitro* data from the same paper suggested that the metastatic process is significantly increased when Col 3 level is reduced. The later phenomenon could not be due to TLC engagement because the *in vitro* system did not contain lymphocytes.

To establish whether the p62 plasmid indeed acts via an anti-tumor adaptive immune response, we compared the anti-tumor effect of the p62 plasmid in wt and SCID mice strains**.** Indeed, if the plasmid acts on the cancer cells directly and/or via a mechanism other than an adaptive immune response then the B16 melanoma administered to the two strains would demonstrate the same sensitivity to the plasmid. In contrast, if the plasmid acts via stimulating/modulating an adaptive immune response, it would be inactive in SCID mice lacking the lymphocytes. The later turned out to be the case. Although p62 DNA has reduced the growth of the subcutaneous B16 melanoma tumor and increased the rate of survival in the wt animals, the plasmid was completely inactive in SCID mice. Thus, we conclude that p62 DNA acts via adaptive immune system.

Because canine mammary tumors, contrary to human tumors, do not express p62 [25], the plasmid could not act as a classic DNA vaccine encoding p62 as a target tumor-specific antigen. Thus, we hypothesized that p62 DNA enhances the adaptive immune response to tumor antigens other than p62. To test this hypothesis we conducted an adoptive T-cell transfer experiment. T-lymphocytes were isolated from mice challenged with transplantable tumor models and, propagated *ex vivo,* were administered to animals bearing the same tumors. Obviously an absolute majority of transferred T-cells were targeted against tumor antigens other than p62. This led to a significant but incomplete reduction of the number of tumor lesions in the lungs. Now we could test if supplementing T-cell transfer with p62 plasmid would lead to a greater protective effect that T-cell transfer alone. The murine cancer model is not p62 negative. However, we administered p62 DNA at late time points, so the animals did not have enough time to develop a protective anti-p62 immune response which could influence the formation of tumor lesions in lungs. According to our experience, this type of antigenic affect would take at least 3 weeks. Nevertheless, we observed that treatment with the p62 plasmid enhanced effects of adaptive T-cell transfer. We interpret this result as the ability of injected p62 plasmid to enhance the anti-cancer effect of T-lymphocytes targeted against non-p62 antigenic cancer epitopes. The fact that the p62 plasmid acts via an adaptive immune response well corresponds to the observations that the plasmid restores sensitivity to chemotherapy. Despite the fact that originally chemotherapeutic agents were selected based their ability to kill rapidly dividing cells, it turned out that many of them act via the immune system (e.g. stimulating immune- presenting cell death or regulating T-regs) [43, 44]. Thus, the result of chemotherapy is a lymphocyte attack on cancer cells. Creating a tumor microenvironment favorable for active TILs makes p62 DNA an equally promising adjuvant for immune-, chemo- and radiation therapies because all of them involve immune response.

## CONCLUSIONS

While acknowledging that a sizable clinical study is necessary to evaluate if the results we observed on a limited sample size are representative for dogs of different ages and breeds, this pilot investigation lead us to propose that p62 DNA treatment can reprogram tumor stroma. The plasmid can be used as an adjuvant for cancer therapies directly and/or indirectly acting through an immune response (such as chemo- and immunotherapies).

## MATERIALS AND METHODS

### p62 DNA plasmid

Human p62 (Sqstm1, isoform 1) – encoding plasmid was described elsewhere [37] and produced endotoxin Free- GMP grade by the Aldevron (ND, USA). Endotoxin-free pcDNA3.1 plasmid was prepared by alkaline lysis using Endo Free Plasmid Kit (Qiagen).

### Dogs patients and treatment

Assessment of the therapeutic effect was performed in veterinary clinic of the University of Camerino (Italy). A total of six dogs, all females, of different breeds and ages were enrolled in the study (Table 1). All of them had histologically confirmed diagnosis of mammary carcinoma with WHO stages I-III, progressive disease. p62 DNA was administered i.m. once a weak at the doses of 0.75, 1.5 mg for 3–9 weeks (Table 1). During the treatment, blood samples were collected, and the tumors sizes / volumes were weekly measured with calipers according to formula π /6 × L × W × H. The weight and overall well-being of patients were monitored. All the treatments were performed with full consent of the owners.

### Tumor specimens and immunohistochemistry

Biopsies (Trucut) were performed in all dogs before treatment to establish initial diagnosis. In 5 out of 6 patients, a second biopsy, along with samples from resected tumors (mastectomy) were collected. Patients # 5 had no mastectomy (see Text). The samples, fixed in 10% neutral buffered formalin, were subjected to histological and immunohistochemical analysis. For each sample 4 μm-thick sections were obtained; one section was stained with haematoxylin-eosin, the other was used for the immunohistochemical analysis. The samples were histologically classified and graded according to criteria of Goldschmidt et al. [32].

For immunohistochemistry, sections were mounted on Superfrost^®^Plus slides and an avidin–biotin–peroxidase-complex (ABC) technique with diaminobenzidine as the chromogen was performed to evaluate the antigen expression. To investigate stromal and ECM responses after p62 plasmid administration, sections were incubated with the following antibodies: mouse monoclonal anti-alpha SMA (1:50; Sigma Aldrich, S. Luis, Missuri USA); rabbit polyclonal anti Collagen type 1 (1:75; Novocastra, Newcastle, UK); mouse monoclonal anti- Collagen type III (Abcam, Cambridge, UK). Sections were counterstained in Mayer’s haematoxylin.

### Mice and tumor growth assessment

C57BL/6J mice were control (wt) animals, and immunodeficient B6. CB17- *Prkdc^scid^*/SzJ (C57BL/6 scid). All mice (females 6–8 weeks,18–20 g) were from Jackson Lab (Bar Harbor, ME, USA). Mice (15 per group) were inoculated with B16 melanoma (3 × 10^5^ in 0.1 ml of PBS) s.c. in right femurs and injected with p62 plasmid (300 ug/mouse i.m in 0.1 ml of saline), or saline (0.1 ml) as a control on days 1, 8, 15 after tumor inoculation. Our previous experiments demonstrated that sham vector (pcDNA3.1) had no effect on tumor growth (FMV unpublished observation). Tumor growth was monitored every other day by a caliper as indicated above. Statistical analysis was performed by two-way ANOVA with Bonferroni post-tests.

### Tumor-draining lymph nodes (TDLN) T cells for adoptive immunotherapy

C57BL/6J mice were inoculated subcutaneously with 1 × 10^6^ MCA205 fibrosarcoma cells in both flanks. Twelve days later, inguinal TDLNs (tumor-draining lymph node) were harvested, and single-cell suspensions were prepared and culture -activated with anti-CD3 antibody and IL-2 as described [45]. Four days later, TDLN cells were resuspended in Hanks´ balanced salt solution (HBSS) for adoptive immunotherapy [46, 47]. Therapeutic efficacy of transferred T effector cells was assessed in the treatment of 9-day established MCA205 pulmonary metastases by intravenous injection of 5 × 10^6^ culture- activated T cells to each mouse and / or p62 plasmid (300 ug/mouse i.m) on days 9 and 14. To improve the therapeutic efficacy of transferred T cells, tumor-bearing mice were pretreated intravenously with cyclophosphamide (100 mg/kg) 1 day before T cells infusion [48].

### Assessment of antitumor effect

For establishment of pulmonary metastases, C57BL/6J mice were injected intravenously with 3 × 10^5^ MCA205 suspended in 200 ul of HBSS. On day 26 after inoculation, MCA205 tumor–bearing lungs were counterstained with India ink and were enumerated. Lungs with more than 250 nodules were assigned >250 as the maximum number that can be counted reliably.

## Supplementary Material

Supplementary Figure 1
