# Supplementary material for: p62-DNA-encoding plasmid reverts tumor grade, changes tumor stroma, and enhances anticancer immunity
Source: Aging (Albany NY). 2019 Nov 21;11(22):10711–22. doi: 10.18632/aging.102486 (PMC6914433; doi:10.18632/aging.102486)
Supplement: Supplementary Figure 1 [file aging-11-102486-s001..pdf]

SUPPLEMENTARY FIGURE

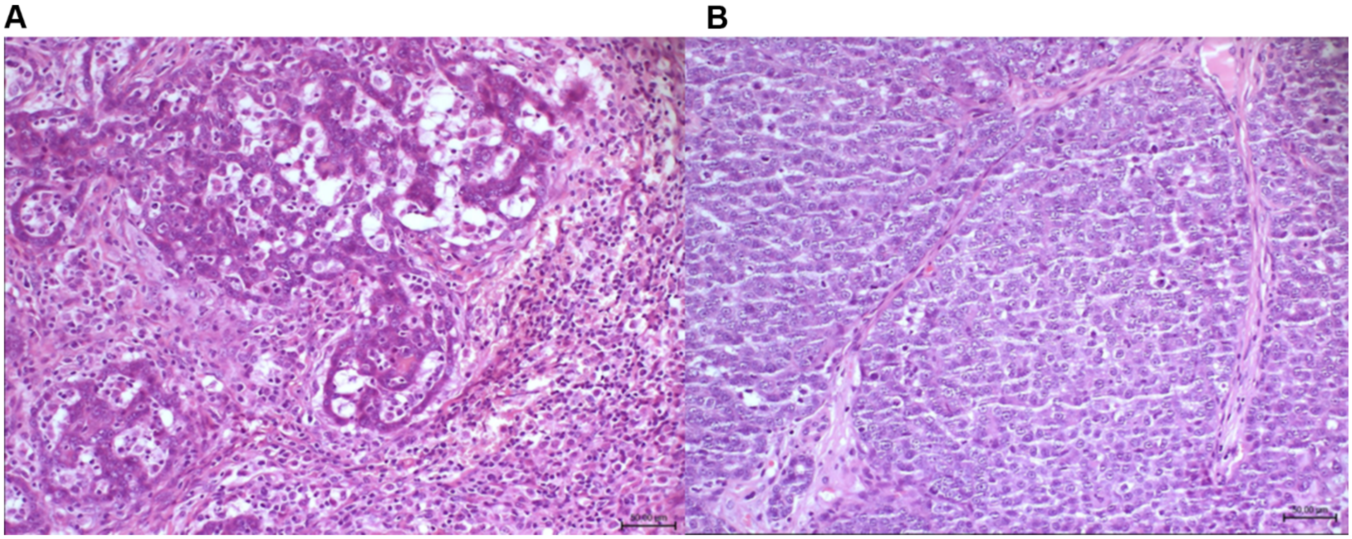

Supplementary Figure 1. Histological examination of solid carcinomas (**A** and **B**) 7 weeks after treatment with DNA plasmids. (**A**) p62 DNA (Injection dose-1.5mg: #5 injection). (**B**) pcDNA3.1 sham vector (Injection dose-1.5mg: #5 injection). Bars, 50 µm.
